# Clinical Presentation and Antibiotic Susceptibility of Contact Lens Associated Microbial Keratitis

**DOI:** 10.1155/2015/152767

**Published:** 2015-12-03

**Authors:** Hesam Hedayati, Mahboubeh Ghaderpanah, Seyed Ahmad Rasoulinejad, Mohammad Montazeri

**Affiliations:** ^1^Department of Ophthalmology, Ahvaz Jundishapur University of Medical Sciences, Ahvaz, Iran; ^2^Department of Ophthalmology, Babol University of Medical Sciences, Babol, Iran; ^3^Young Researchers Club, Islamic Azad University, Babol Branch, Babol, Iran

## Abstract

*Introduction.* In recent years, the number of contact lens wearers has dramatically increased in Iran, particularly in youngsters. The purpose of current study was to assess the clinical presentation and antibiotic susceptibility of contact lens related microbial keratitis in Ahvaz, southwest of Iran.* Methodology.* A cross-sectional investigation of 26 patients (33 eyes) with contact lens induced corneal ulcers who were admitted to Imam Khomeini Hospital, Ahwaz City, from June 2012 to June 2013 was done. In order to study microbial culture and susceptibility of corneal ulcers, all of them were scraped.* Results.* Eight samples were reported as sterile.* Pseudomonas aeruginosa* (80%) in positive cultures was the most widely recognized causative organism isolated. This is followed by* Staphylococcus aureus* 12% and* Enterobacter* 8%. The results showed that 84% of the microorganism cases were sensitive to ciprofloxacin, while imipenem, meropenem, and ceftazidime were the second most effective antibiotics (76%).* Conclusion.* Results of current study show the importance of referring all contact lens wearers with suspected corneal infection to ophthalmologists for more cure. The corneal scraping culture and contact lens solution should be performed to guide antibiotic therapy.

## 1. Introduction

Microbial keratitis (corneal ulcer) is an infective methodology of the cornea and is a conceivable sight-threatening condition and serious visual impairments [1]. It may cause a noteworthy public health issue. Untreated or severe keratitis may impact on perforation and endophthalmitis [2, 3]. Contact lenses are a key reason for microbial keratitis in the developed nations where they are broadly accessible, mainly in young adults [4]. Contact lens related keratitis is a serious impediment of contact lens wear, with nearly one out of five hospitalized cases needing corneal transplantation [5]. The incidence of contact lens related microbial keratitis has been enhanced in developed countries [3, 6]. The incidence of contact lens related keratitis is wildly different around the world and over time, which includes the varying contact lens solution market and local environmental problems, such as water storage and disinfection [7].

Though microbial keratitis is an uncommon difficulty of contact lens wear, the severity of infection, the number of lens wearers, and the risk to vision prepare important causes to assess this difficulty of contact lens wearer [8]. Lately, the number of contact lens wearers has been enhanced significantly in Iran, particularly in youngsters [9]. Therefore, the aim of current study was to assess the frequency and microbiological profile of keratitis between patients wearing all current types of contact lenses designed for both extended and daily wear, referred to Imam Khomeini Hospital, Ahwaz City, southwest of Iran, in a one-year period.

## 2. Methodology

### 2.1. Study Design and Patients

A cross-sectional investigation was conducted for all patients with contact lens induced corneal ulcers who were admitted to Imam Khomeini Hospital, Ahvaz City, southwest of Iran, during one year (from June 2012 to June 2013). Contact lens related microbial keratitis is described as a supportive corneal infiltrate and overlying epithelial defect with latest history of contact lens use, with or without hypopyon.

Patients were omitted from the study if they have an earlier history of anterior segment surgery, utilization of any topical ocular medications, or treatment for ocular surface disease. Moreover, noninfectious corneal ulcerations such as Mooren's ulcer, marginal keratitis, sterile neurotropic ulcers, and ulcers related with autoimmune disease were excluded. Every patient was examined by an ophthalmologist at the slit lamp. Clinical features (including redness, foreign body sensation, pain, chemosis, blurred vision, epiphora, discomfort, discharge, photophobia, swelling, and ocular redness) were considered and a drawing was prepared for records of patient.

All patients wore soft contact lenses, utilizing either disposable extended wear lenses or conventional daily soft contact lenses. The sterilization regimen involved hydrogen peroxide or no hygiene regimen.

### 2.2. Sampling, Culture, and Susceptibility Tests

After a detailed examination of ocular, all suspected infectious corneal ulcers were scraped to study microbial culture and susceptibility before starting the treatment.

After instillation of 0.5% proparacaine hydrochloride, two slides were done by an ophthalmologist to perform the direct microscopic examination utilizing a sterile 21-gauge needle or flame-sterilized Kimura spatula, from the leading edge and the bed of the ulcer.

The achieved material was spread onto marked slides for gram and Giemsa stains. In addition, for growth of bacterial and fungal colonies, the material was placed onto the agar culture plates' surfaces using cotton swab applicators.

In our ophthalmology ward, the usual practice for patients with corneal ulcers is to do smears for gram stain and after that culture the samples in three diverse mediums: chocolate agar, blood agar, and sabouraud agar (for fungal infections). A single colony of a virulent organism or at least three colonies of an organism that generally is not noted to be greatly pathogenic on the surface of ocular (e.g., coagulase negative* Staphylococcus*) were noted to be positive cultures. If the culture was recorded as positive for bacterial keratitis after 72 h, the antibiotics resistance was measured within the Mueller-Hinton media and interpreted based on the guiding principle established by the National Committee on Clinical Laboratory Standards [10, 11].

### 2.3. Visual Acuity

Any decrease in vision was examined using Snellen letter charts acuity. Decrease in vision was determined compared to the unaffected eye. If both eyes were affected, computing the amount of vision loss was performed by utilizing the worse eye and a standard reference of 6/6 Snellen acuity. In the same way, when amblyopia was in the unaffected eye, a standard reference of 6/6 was utilized for comparison. Visual acuity was classified as no light perception (NLP), hand motion (HM), counting fingers (CF), loss of 2 or more lines, or no loss of vision [9].

### 2.4. Size of the Corneal Ulcer

When the microbial keratitis impacts the visual axis, ulcers are noted to be central. Ulcers are considered as peripheral ulcers, if they were lateral to the midpoint of an imaginary line between the visual axis and limbus [9].

The size of ulcer provides an estimate of the ulcer area to ease statistical analysis and computed as in the following: size of ulcer = length × breadth/mm^2^.

### 2.5. Disease and Hospitalization Duration

Duration of disorders was described by the number of days that symptoms including discomfort, blurred vision, photophobia, redness, discharge, and swelling were experienced. The period of stay in hospital is defined as hospitalization duration [9].

### 2.6. Outcome

Cases were considered to have an “excellent” clinical result if corneal treatment was not related to a visual loss or no scar, “good” results when they lost less than 2 lines of visual acuity or mild to moderate scar, and “poor” results when visual loss was superior to 2 lines or when a major complication happened or when they experienced penetrating keratoplasty [9].

### 2.7. Statistical Analysis

The SPSS software of windows (version 18.0, SPSS Inc., Chicago, IL, USA) was used for statistical analysis.

## 3. Results

A total of 26 subjects (33 eyes) were enrolled into the research. Out of 26, 2 (7.7%) were males and 24 (92.3%) were females. The age of the subjects was in the range of 16 to 41 years (average age 23.88 ± 9.41 years). In these subjects, the average delay between the beginning the signs and the first examination was 48 hours.

Keratitis involved the right eye and the left eye in 9 (34.6%) and 10 (38.5%) of patients, respectively. Seven cases (26.9%) had bilateral infection.

All 26 cases were contact lens wearers, and there were 6 patients (23.1%) and 20 patients (76.9%) for therapeutic contact lens use and cosmetic lens use, respectively. All cosmetic lenses were conventional daily and therapeutic contact lenses were disposable extended wear.

Overnight lens use was considered in six patients. Five of the 20 cosmetic contact lens wearers were utilizing lenses of another person at the time of the infectious event, and 21 cases (80.8%) selected and wore their lenses without any ophthalmology consultation.

Fifteen (57.7%) cases were daily lenses wearers; however 11 cases (42.3%) wore extended contact lenses. Hydrogen peroxide was used by nine (34.6%) cases for disinfection of contact lenses, while 17 (65.4%) cases utilized no disinfection procedure.

**Figure 1:**
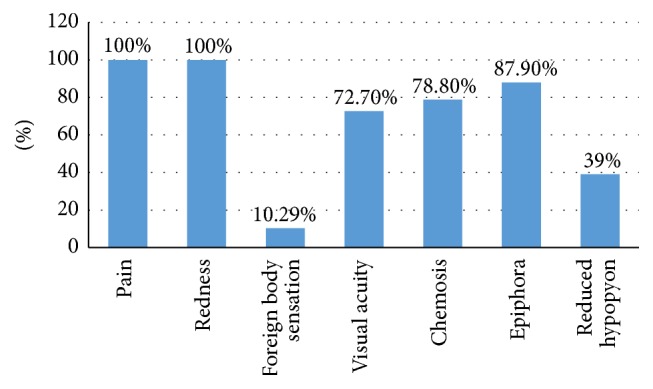
The frequency of symptoms and signs in patients with microbial keratitis.

Symptoms and signs were characterized in Figure 1. Pain and redness were the most prevalent clinical signs that were observed and reported for all subjects.

**Figure 2:**
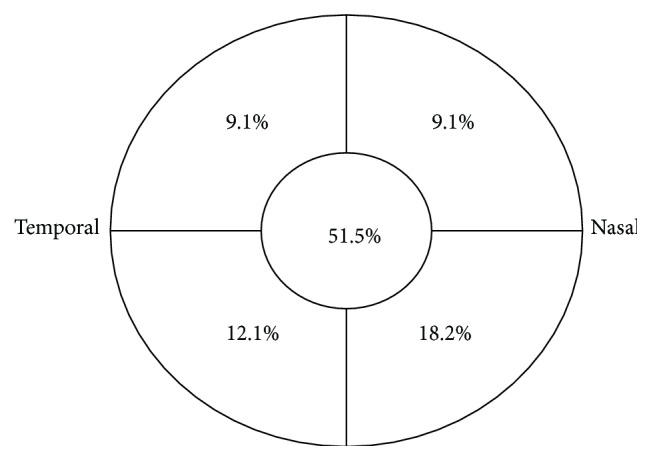
Location of the principal corneal infiltrate (6.1% of the infiltrates were diffuse).

As presented in Figure 2, the infiltrates position was distributed; 6.1% of the infiltrates were diffuse (2 eyes). A predominance of central localization (51.5%) was observed. The average of ulcer size was 4.12 ± 3.76 mm^2^. The corneal ulcer size was less than 3 mm^2^ for 13 eyes (39.4%), 3–6 mm^2^ for 11 (33.3%), and greater than 6 mm^2^ for 9 (27.3%).

Visual acuity was HM in 33.3% of the subjects, FC in 24.2%, and >1/10 in 42.4%.

Initial treatment of all cases of microbial keratitis was carried out with topical levofloxacin. Eight samples were reported as sterile.* Pseudomonas aeruginosa* (80%) in positive cultures was the most widely recognized causative organism isolated. This is followed by* Staphylococcus aureus* 12% and* Enterobacter* 8%. The results of antibiogram in Table 1 indicate that 84% of the microorganism cases were sensitive to ciprofloxacin, while imipenem, meropenem, and ceftazidime were the second most effective antibiotics (76%).

There were not any other risk factors leading to microbial keratitis among 6 patients who wear contact lens for therapeutic reason.

Out of these patients, 57.7% of treated outpatients, 34.6% of them were admitted and 7.7% required surgery interventions. Average treatment period was 31 ± 6 days and 84 ± 12 days in outpatient and inpatient subjects, respectively. According to the obtained results, treatment outcomes were excellent in 24.2%, good in 45.5%, and poor in 30.3%.

## 4. Discussion

Corneal ulcers without any doubt are the most devastating difficulty of soft contact lens utilization. A total of 26 cases (33 eyes) were enrolled in current research. Most of subjects (92.3%) were females with the average age of 23.88 ± 9.41 years.

In a retrospective investigation conducted by Mela et al. [12], 23 patients admitted with contact lens related corneal ulcers were reported during a 43-month period. All of the cases were utilizing soft contact lenses for 3 days to 20 years and most cases were young women. Malaysian National Eye Database Study Group [13] reported 202 patients with the contact lens related corneal ulcers (CLRCU) registry and an average age of 26.7 years (71.8% female), during 2007-2008. All subjects wore soft contact lens.

Assessment of 56 ulcerative keratitis cases corresponding with contact lens wear indicated contact lens related ulcers were observed in 86% of those wearing soft lenses [14]. Benhmidoune et al. [15] conducted a descriptive study of 51 cases presenting with contact lens associated corneal ulcers to the ophthalmology hospital in Casablanca. The gender ratio of our subjects was 7.5 female to male with the mean age of 22 years.

In current study 42.3% wore extended contact lenses, while 57.7% were daily lenses wearers. In addition, contact lenses can interfere with typical epithelial proliferation and differentiation that may compromise barrier function. Lenses impact on innate defenses (and microbial virulence) or are more probable to be prevalent with extended wear or overnight recognized risk factors of infection, while daily wear is similarly related to microbial keratitis, and it would be of interest to define when pathogenesis of the disease varies between these lenses wear modalities.

As presented in our investigation, there was a predominance of central localization (51.5%). Several studies suggested that microbial keratitis happening in the peripheral cornea is less clinically severe compared with those happening in the central cornea. When the insult site is close to the limbus, a relatively quick host immune reaction might be expected to restrict the extent of tissue compromise, because of the rather short distance that polymorphonuclear leucocytes and other defensive cellular elements require to transfer from the limbal vessels into the site of tissue insult. It follows that disorders of the central cornea will be less well protected and any advanced type of pathology in this area is probable to progress further before the host immune reaction can dampen the response [16, 17].

A pathogenic causative organism is the initial measurement of disease outcome in contact lens associated microbial keratitis. The pathogen identification is critical as no clinical characteristics of microbial keratitis may be noted as pathognomonic [18]. The kinds of organisms recovered from the corneal scratches are not part of the ocular flora and are broadly dispersed in water, soil, sewage, and gastrointestinal tract of humans and their presence shows that the source of pollution is external in nature [19]. More than half of lenses regularly harbor microorganisms containing potentially pathogenic species; though, the ocular surface tolerates their existence and overcomes potentially disturbing subsequences under normal conditions [20]. We recognized that* Pseudomonas aeruginosa* were the main causative agents of contact lens associated microbial keratitis, accounting for nearly half of the culture-proven infections. Earlier investigations indicated that* Pseudomonas aeruginosa* is the most commonly recovered causative organism of contact lens related disease, followed by gram-positive bacteria,* Acanthamoeba,* and fungi [12–15, 21, 22]. Goh et al. [13] reported that* Pseudomonas* (79.7% of bacterial cases) was the most common causative organism in Malaysia. In Mela et al. study [12], the most frequent isolated pathogen (60%) was* Pseudomonas aeruginosa*. Benhmidoune et al. [15] reported that 47.8% of their studied subjects had positive corneal bacterial cultures. Pathogens recognized were* Staphylococcus aureus*,* Pseudomonas aeruginosa*, and* Acanthamoeba*. In Galentine et al. study [14], the most common isolate was* Pseudomonas* happening in 13 (23%) of the 56 patients.* Staphylococcus* species were the next most common, happening in 11 (20%) of the 56 subjects.

*Pseudomonas aeruginosa* have a tendency to adhere to the contact lens surface and is transferred over scratched corneal epithelium penetrating the cornea's deeper layers and leading corneal ulcers. Permanent blindness can be caused by a severe infection lead. The lens, ocular environment, and storage case may offer an appropriate survival niche for this environmental organism.* Pseudomonas aeruginosa* can adhere to and colonize lens materials during wear and survive in contact lens storage cases [20].

## 5. Conclusion

Results of current study propose the importance of referring all contact lens wearers with suspected corneal infection to ophthalmologists for more cure and the corneal scraping culture and contact lens solution should be performed to guide antibiotic therapy.

Inappropriate lens wear and care and absence of awareness of the importance of aftercare visits have been known as risk factors for corneal ulcer among contact lens wearers. Teaching and enhancing awareness of sufficient lens care and disinfection practices, counseling with an ophthalmologist, and regular replacement of contact lens storage cases would be really helpful to decrease this risk of microbial keratitis. The daily-disposable lenses use should be encouraged since it has been proposed to decrease the risk of developing ulcer when presented at the disinfectant step. Moreover, contact lens wearers ought to be encouraged to prepare their lenses from eye health suppliers.

## Figures and Tables

**Table 1:** Antibiotic sensitivity and resistance pattern of microorganism isolated from corneal ulcers in patients with microbial keratitis.

	*Pseudomonas aeruginosa*		*Staphylococcus aureus*		*Enterobacter*	
	Sensitive	Resistant	Sensitive	Resistant	Sensitive	Resistant
	Number (%)	Number (%)	Number (%)	Number (%)	Number (%)	Number (%)
Penicillin	0 (0)	20 (100)	0 (0)	3 (100)	0 (0)	2 (100)
Ciprofloxacin	19 (95)	1 (5)	0 (0)	3 (100)	2 (100)	0 (0)
Gentamycin	0 (0)	20 (100)	3 (100)	0 (0)	2 (100)	0 (0)
Amikacin	10 (50)	10 (50)	0 (0)	3 (100)	2 (100)	0 (0)
Cephalexin	0 (0)	20 (100)	0 (0)	3 (100)	0 (0)	2 (100)
Ceftazidime	17 (85)	3 (15)	0 (0)	3 (100)	2 (100)	0 (0)
Cefixime	0 (0)	20 (100)	0 (0)	3 (100)	2 (100)	0 (0)
Imipenem	17 (85)	3 (15)	0 (0)	3 (100)	2 (100)	0 (0)
Meropenem	17 (85)	3 (15)	0 (0)	3 (100)	2 (100)	0 (0)
